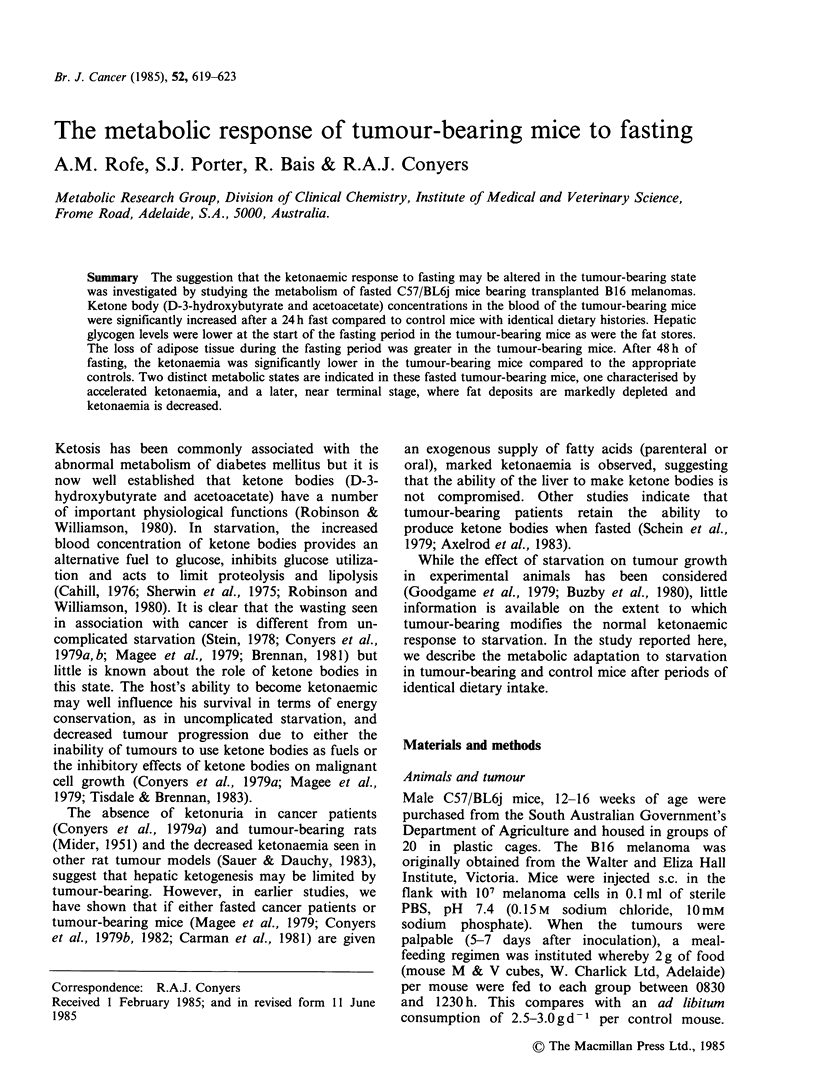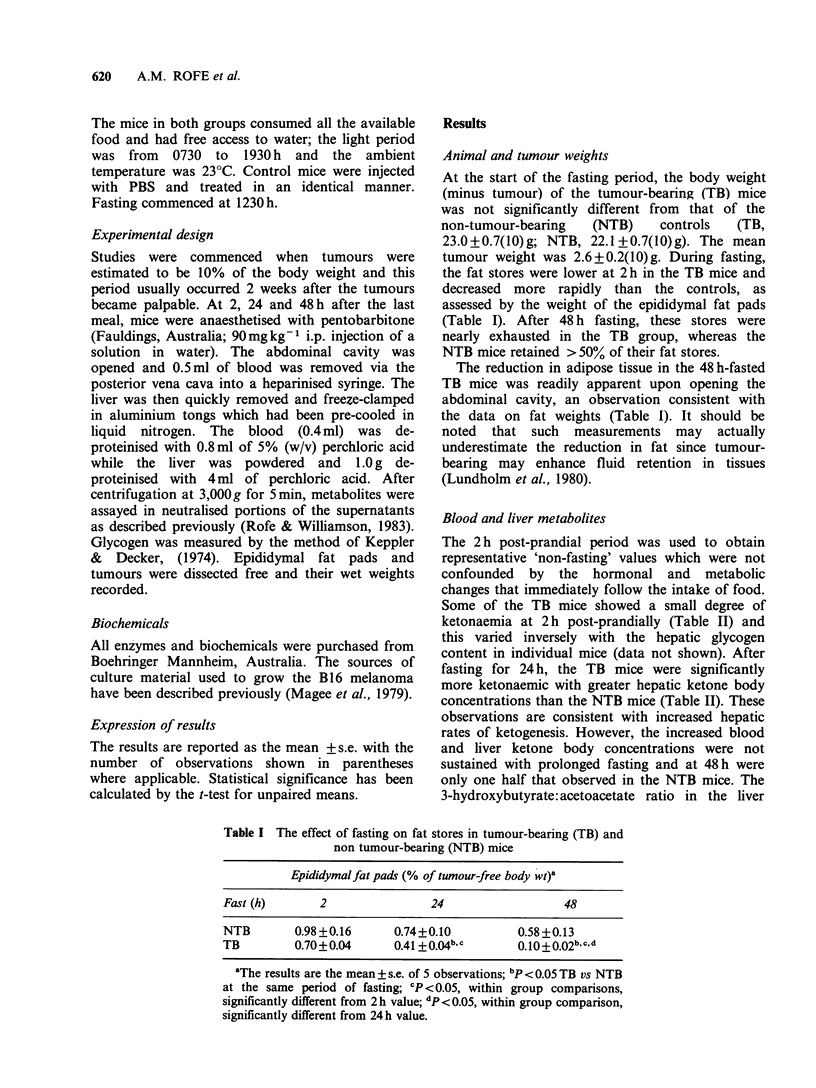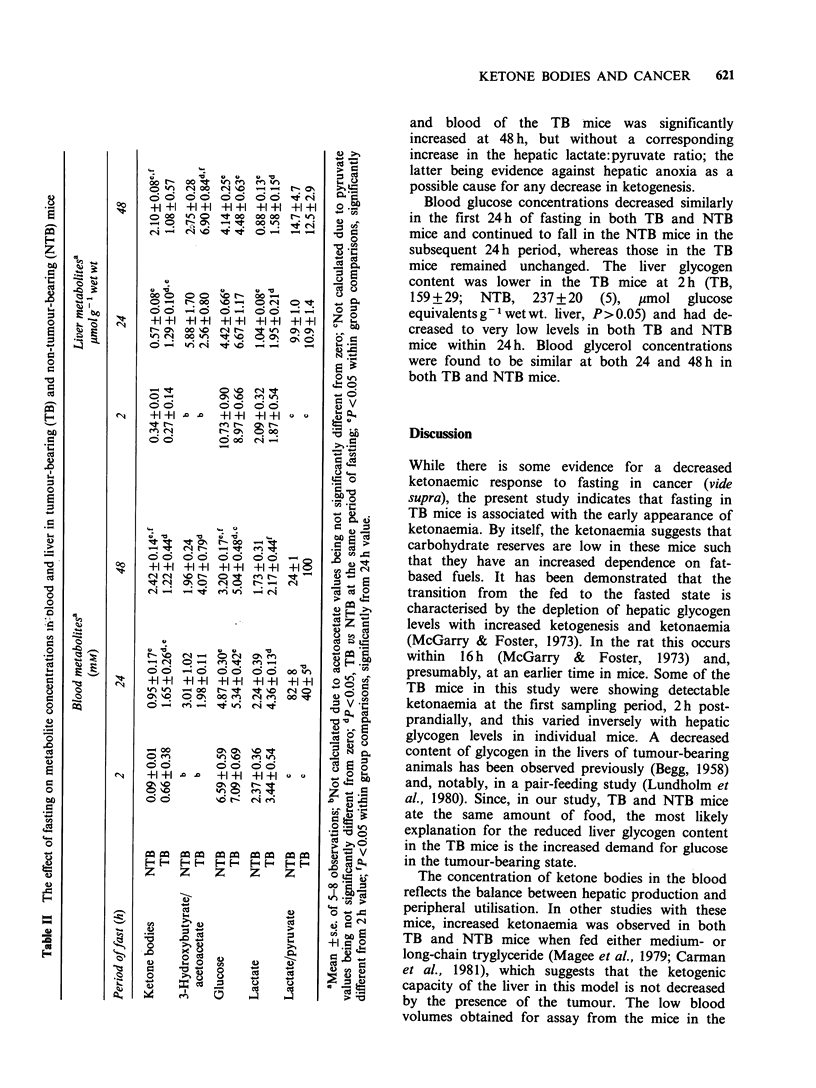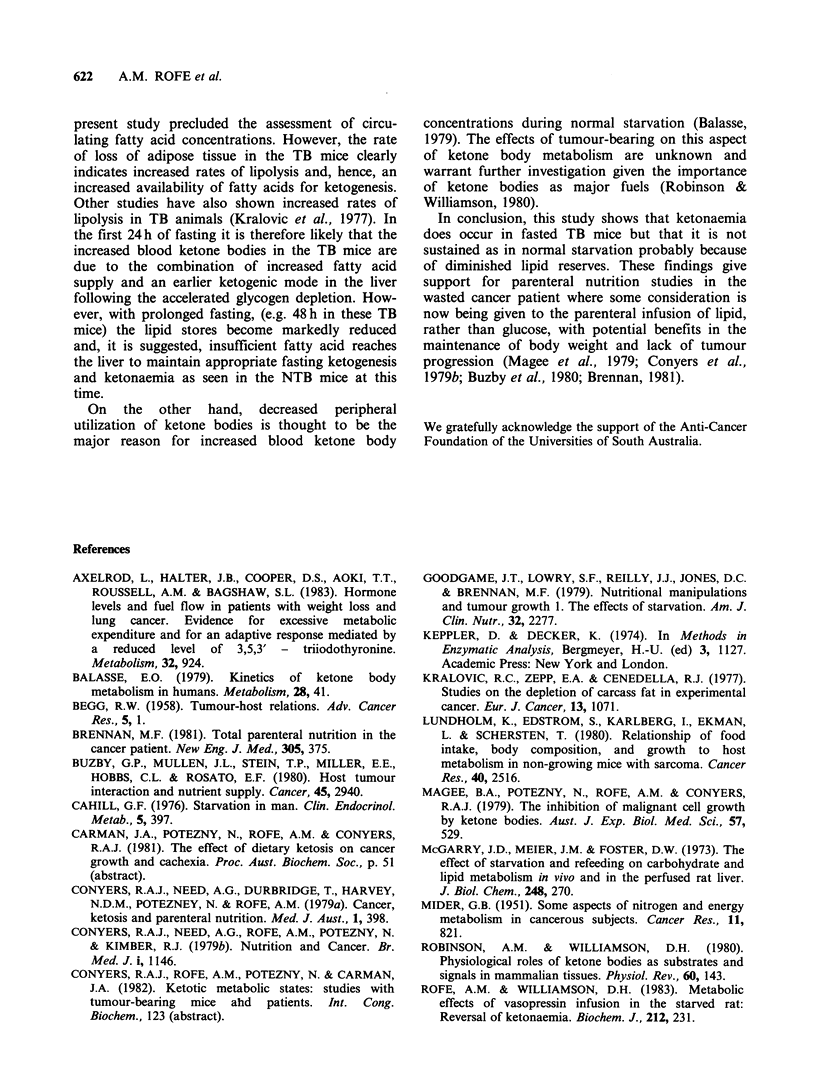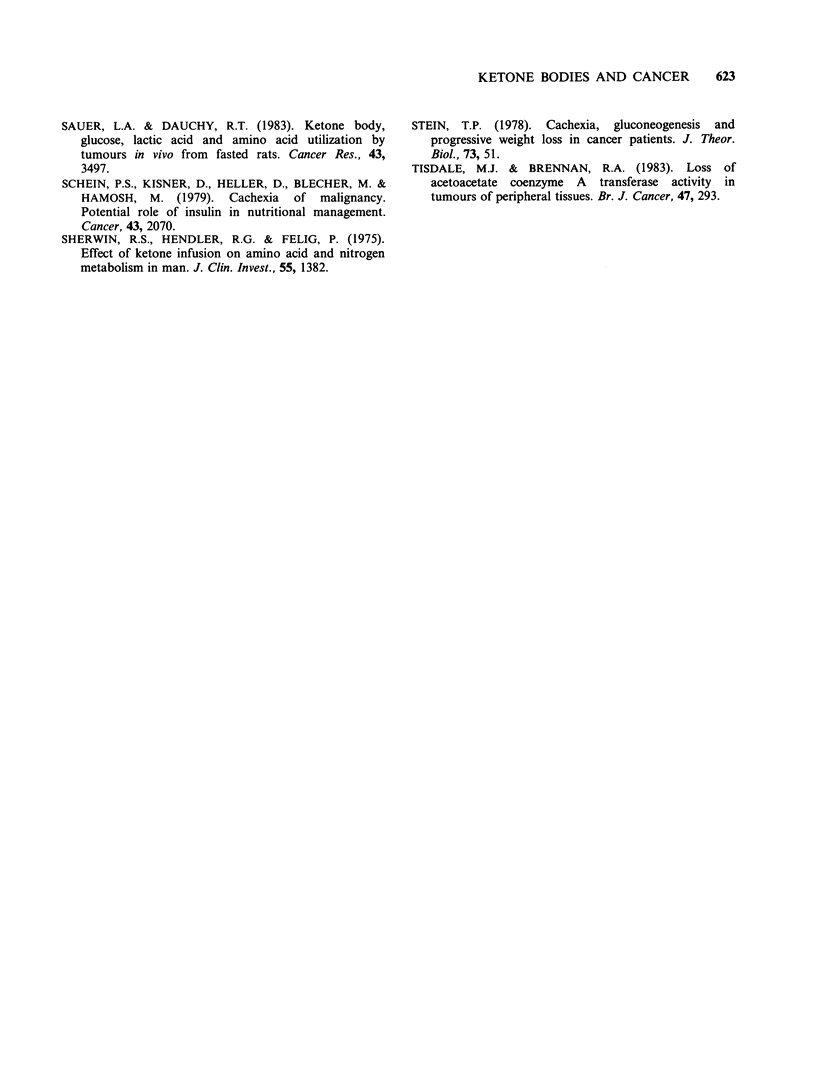# The metabolic response of tumour-bearing mice to fasting.

**DOI:** 10.1038/bjc.1985.235

**Published:** 1985-10

**Authors:** A. M. Rofe, S. J. Porter, R. Bais, R. A. Conyers

## Abstract

The suggestion that the ketonaemic response to fasting may be altered in the tumour-bearing state was investigated by studying the metabolism of fasted C57/BL6j mice bearing transplanted B16 melanomas. Ketone body (D-3-hydroxybutyrate and acetoacetate) concentrations in the blood of the tumour-bearing mice were significantly increased after a 24 h fast compared to control mice with identical dietary histories. Hepatic glycogen levels were lower at the start of the fasting period in the tumour-bearing mice as were the fat stores. The loss of adipose tissue during the fasting period was greater in the tumour-bearing mice. After 48 h of fasting, the ketonaemia was significantly lower in the tumour-bearing mice compared to the appropriate controls. Two distinct metabolic states are indicated in these fasted tumour-bearing mice, one characterised by accelerated ketonaemia, and a later, near terminal stage, where fat deposits are markedly depleted and ketonaemia is decreased.


*Br. J. Cancer* (1985), **52**, 619–623

### The metabolic response of tumour-bearing mice to fasting

A.M. Rofe, S.J. Porter, R. Bais & R.A.J. Conyers

*Metabolic Research Group, Division of Clinical Chemistry, Institute of Medical and Veterinary Science, Frome Road, Adelaide, S.A., 5000, Australia.*

**Summary** The suggestion that the ketonaemic response to fasting may be altered in the tumour-bearing state was investigated by studying the metabolism of fasted C57/BL6j mice bearing transplanted B16 melanomas. Ketone body (D-3-hydroxybutyrate and acetoacetate) concentrations in the blood of the tumour-bearing mice were significantly increased after a 24 h fast compared to control mice with identical dietary histories. Hepatic glycogen levels were lower at the start of the fasting period in the tumour-bearing mice as were the fat stores. The loss of adipose tissue during the fasting period was greater in the tumour-bearing mice. After 48 h of fasting, the ketonaemia was significantly lower in the tumour-bearing mice compared to the appropriate controls. Two distinct metabolic states are indicated in these fasted tumour-bearing mice, one characterised by accelerated ketonaemia, and a later, near terminal stage, where fat deposits are markedly depleted and ketonaemia is decreased.

Ketosis has been commonly associated with the abnormal metabolism of diabetes mellitus but it is now well established that ketone bodies (D-3-hydroxybutyrate and acetoacetate) have a number of important physiological functions (Robinson & Williamson, 1980). In starvation, the increased blood concentration of ketone bodies provides an alternative fuel to glucose, inhibits glucose utilization and acts to limit proteolysis and lipolysis (Cahill, 1976; Sherwin *et al.*, 1975; Robinson and Williamson, 1980). It is clear that the wasting seen in association with cancer is different from uncomplicated starvation (Stein, 1978; Conyers *et al.*, 1979*a,b*; Magee *et al.*, 1979; Brennan, 1981) but little is known about the role of ketone bodies in this state. The host's ability to become ketonaemic may well influence his survival in terms of energy conservation, as in uncomplicated starvation, and decreased tumour progression due to either the inability of tumours to use ketone bodies as fuels or the inhibitory effects of ketone bodies on malignant cell growth (Conyers *et al.*, 1979*a*; Magee *et al.*, 1979; Tisdale & Brennan, 1983).

The absence of ketonuria in cancer patients (Conyers *et al.*, 1979*a*) and tumour-bearing rats (Mider, 1951) and the decreased ketonaemia seen in other rat tumour models (Sauer & Dauchy, 1983), suggest that hepatic ketogenesis may be limited by tumour-bearing. However, in earlier studies, we have shown that if either fasted cancer patients or tumour-bearing mice (Magee *et al.*, 1979; Conyers *et al.*, 1979*b*, 1982; Carman *et al.*, 1981) are given an exogenous supply of fatty acids (parenteral or oral), marked ketonaemia is observed, suggesting that the ability of the liver to make ketone bodies is not compromised. Other studies indicate that tumour-bearing patients retain the ability to produce ketone bodies when fasted (Schein *et al.*, 1979; Axelrod *et al.*, 1983).

While the effect of starvation on tumour growth in experimental animals has been considered (Goodgame *et al.*, 1979; Buzby *et al.*, 1980), little information is available on the extent to which tumour-bearing modifies the normal ketonaemic response to starvation. In the study reported here, we describe the metabolic adaptation to starvation in tumour-bearing and control mice after periods of identical dietary intake.

Correspondence: R.A.J. Conyers
Received 1 February 1985; and in revised form 11 June 1985

### Materials and methods

### *Animals and tumour*

Male C57/BL6j mice, 12–16 weeks of age were purchased from the South Australian Government's Department of Agriculture and housed in groups of 20 in plastic cages. The B16 melanoma was originally obtained from the Walter and Eliza Hall Institute, Victoria. Mice were injected s.c. in the flank with $10^7$ melanoma cells in 0.1 ml of sterile PBS, pH 7.4 (0.15 M sodium chloride, 10 mM sodium phosphate). When the tumours were palpable (5–7 days after inoculation), a meal-feeding regimen was instituted whereby 2 g of food (mouse M & V cubes, W. Charlick Ltd, Adelaide) per mouse were fed to each group between 0830 and 1230 h. This compares with an *ad libitum* consumption of 2.5–3.0 g $d^{-1}$ per control mouse.

© The Macmillan Press Ltd., 1985

The mice in both groups consumed all the available food and had free access to water; the light period was from 0730 to 1930 h and the ambient temperature was 23°C. Control mice were injected with PBS and treated in an identical manner. Fasting commenced at 1230 h.

### *Experimental design*

Studies were commenced when tumours were estimated to be 10% of the body weight and this period usually occurred 2 weeks after the tumours became palpable. At 2, 24 and 48 h after the last meal, mice were anaesthetised with pentobarbitone (Fauldings, Australia; 90 mg kg$^{-1}$ i.p. injection of a solution in water). The abdominal cavity was opened and 0.5 ml of blood was removed via the posterior vena cava into a heparinised syringe. The liver was then quickly removed and freeze-clamped in aluminium tongs which had been pre-cooled in liquid nitrogen. The blood (0.4 ml) was deproteinised with 0.8 ml of 5% (w/v) perchloric acid while the liver was powdered and 1.0 g deproteinised with 4 ml of perchloric acid. After centrifugation at 3,000 *g* for 5 min, metabolites were assayed in neutralised portions of the supernatants as described previously (Rofe & Williamson, 1983). Glycogen was measured by the method of Keppler & Decker, (1974). Epididymal fat pads and tumours were dissected free and their wet weights recorded.

### *Biochemicals*

All enzymes and biochemicals were purchased from Boehringer Mannheim, Australia. The sources of culture material used to grow the B16 melanoma have been described previously (Magee *et al.*, 1979).

### *Expression of results*

The results are reported as the mean ±s.e. with the number of observations shown in parentheses where applicable. Statistical significance has been calculated by the *t*-test for unpaired means.

### Results

### *Animal and tumour weights*

At the start of the fasting period, the body weight (minus tumour) of the tumour-bearing (TB) mice was not significantly different from that of the non-tumour-bearing (NTB) controls (TB, 23.0±0.7(10) g; NTB, 22.1±0.7(10) g). The mean tumour weight was 2.6±0.2(10) g. During fasting, the fat stores were lower at 2 h in the TB mice and decreased more rapidly than the controls, as assessed by the weight of the epididymal fat pads (Table I). After 48 h fasting, these stores were nearly exhausted in the TB group, whereas the NTB mice retained >50% of their fat stores.

The reduction in adipose tissue in the 48 h-fasted TB mice was readily apparent upon opening the abdominal cavity, an observation consistent with the data on fat weights (Table I). It should be noted that such measurements may actually underestimate the reduction in fat since tumour-bearing may enhance fluid retention in tissues (Lundholm *et al.*, 1980).

### *Blood and liver metabolites*

The 2 h post-prandial period was used to obtain representative 'non-fasting' values which were not confounded by the hormonal and metabolic changes that immediately follow the intake of food. Some of the TB mice showed a small degree of ketonaemia at 2 h post-prandially (Table II) and this varied inversely with the hepatic glycogen content in individual mice (data not shown). After fasting for 24 h, the TB mice were significantly more ketonaemic with greater hepatic ketone body concentrations than the NTB mice (Table II). These observations are consistent with increased hepatic rates of ketogenesis. However, the increased blood and liver ketone body concentrations were not sustained with prolonged fasting and at 48 h were only one half that observed in the NTB mice. The 3-hydroxybutyrate:acetoacetate ratio in the liver

**Table I** The effect of fasting on fat stores in tumour-bearing (TB) and non tumour-bearing (NTB) mice

| | *Epididymal fat pads (% of tumour-free body wt)*[a] | | |
|---|---|---|---|
| *Fast (h)* | *2* | *24* | *48* |
| NTB | 0.98±0.16 | 0.74±0.10 | 0.58±0.13 |
| TB | 0.70±0.04 | 0.41±0.04[b,c] | 0.10±0.02[b,c,d] |

[a]The results are the mean±s.e. of 5 observations; [b]$P<0.05$ TB *vs* NTB at the same period of fasting; [c]$P<0.05$, within group comparisons, significantly different from 2 h value; [d]$P<0.05$, within group comparison, significantly different from 24 h value.

and blood of the TB mice was significantly increased at 48 h, but without a corresponding increase in the hepatic lactate:pyruvate ratio; the latter being evidence against hepatic anoxia as a possible cause for any decrease in ketogenesis.

Blood glucose concentrations decreased similarly in the first 24 h of fasting in both TB and NTB mice and continued to fall in the NTB mice in the subsequent 24 h period, whereas those in the TB mice remained unchanged. The liver glycogen content was lower in the TB mice at 2 h (TB, $159\pm29$; NTB, $237\pm20$ (5), $\mu$mol glucose equivalents $g^{-1}$ wet wt. liver, $P>0.05$) and had decreased to very low levels in both TB and NTB mice within 24 h. Blood glycerol concentrations were found to be similar at both 24 and 48 h in both TB and NTB mice.

### Discussion

While there is some evidence for a decreased ketonaemic response to fasting in cancer (*vide supra*), the present study indicates that fasting in TB mice is associated with the early appearance of ketonaemia. By itself, the ketonaemia suggests that carbohydrate reserves are low in these mice such that they have an increased dependence on fat-based fuels. It has been demonstrated that the transition from the fed to the fasted state is characterised by the depletion of hepatic glycogen levels with increased ketogenesis and ketonaemia (McGarry & Foster, 1973). In the rat this occurs within 16 h (McGarry & Foster, 1973) and, presumably, at an earlier time in mice. Some of the TB mice in this study were showing detectable ketonaemia at the first sampling period, 2 h post-prandially, and this varied inversely with hepatic glycogen levels in individual mice. A decreased content of glycogen in the livers of tumour-bearing animals has been observed previously (Begg, 1958) and, notably, in a pair-feeding study (Lundholm *et al.*, 1980). Since, in our study, TB and NTB mice ate the same amount of food, the most likely explanation for the reduced liver glycogen content in the TB mice is the increased demand for glucose in the tumour-bearing state.

The concentration of ketone bodies in the blood reflects the balance between hepatic production and peripheral utilisation. In other studies with these mice, increased ketonaemia was observed in both TB and NTB mice when fed either medium- or long-chain tryglyceride (Magee *et al.*, 1979; Carman *et al.*, 1981), which suggests that the ketogenic capacity of the liver in this model is not decreased by the presence of the tumour. The low blood volumes obtained for assay from the mice in the present study precluded the assessment of circulating fatty acid concentrations. However, the rate of loss of adipose tissue in the TB mice clearly indicates increased rates of lipolysis and, hence, an increased availability of fatty acids for ketogenesis. Other studies have also shown increased rates of lipolysis in TB animals (Kralovic *et al.*, 1977). In the first 24 h of fasting it is therefore likely that the increased blood ketone bodies in the TB mice are due to the combination of increased fatty acid supply and an earlier ketogenic mode in the liver following the accelerated glycogen depletion. However, with prolonged fasting, (e.g. 48 h in these TB mice) the lipid stores become markedly reduced and, it is suggested, insufficient fatty acid reaches the liver to maintain appropriate fasting ketogenesis and ketonaemia as seen in the NTB mice at this time.

On the other hand, decreased peripheral utilization of ketone bodies is thought to be the major reason for increased blood ketone body concentrations during normal starvation (Balasse, 1979). The effects of tumour-bearing on this aspect of ketone body metabolism are unknown and warrant further investigation given the importance of ketone bodies as major fuels (Robinson & Williamson, 1980).

In conclusion, this study shows that ketonaemia does occur in fasted TB mice but that it is not sustained as in normal starvation probably because of diminished lipid reserves. These findings give support for parenteral nutrition studies in the wasted cancer patient where some consideration is now being given to the parenteral infusion of lipid, rather than glucose, with potential benefits in the maintenance of body weight and lack of tumour progression (Magee *et al.*, 1979; Conyers *et al.*, 1979*b*; Buzby *et al.*, 1980; Brennan, 1981).

We gratefully acknowledge the support of the Anti-Cancer Foundation of the Universities of South Australia.

### References

AXELROD, L., HALTER, J.B., COOPER, D.S., AOKI, T.T., ROUSSELL, A.M. & BAGSHAW, S.L. (1983). Hormone levels and fuel flow in patients with weight loss and lung cancer. Evidence for excessive metabolic expenditure and for an adaptive response mediated by a reduced level of 3,5,3′ – triiodothyronine. *Metabolism*, **32**, 924.

BALASSE, E.O. (1979). Kinetics of ketone body metabolism in humans. *Metabolism*, **28**, 41.

BEGG, R.W. (1958). Tumour-host relations. *Adv. Cancer Res.*, **5**, 1.

BRENNAN, M.F. (1981). Total parenteral nutrition in the cancer patient. *New Eng. J. Med.*, **305**, 375.

BUZBY, G.P., MULLEN, J.L., STEIN, T.P., MILLER, E.E., HOBBS, C.L. & ROSATO, E.F. (1980). Host tumour interaction and nutrient supply. *Cancer*, **45**, 2940.

CAHILL, G.F. (1976). Starvation in man. *Clin. Endocrinol. Metab.*, **5**, 397.

CARMAN, J.A., POTEZNY, N., ROFE, A.M. & CONYERS, R.A.J. (1981). The effect of dietary ketosis on cancer growth and cachexia. *Proc. Aust. Biochem. Soc.*, p. 51 (abstract).

CONYERS, R.A.J., NEED, A.G., DURBRIDGE, T., HARVEY, N.D.M., POTEZNEY, N. & ROFE, A.M. (1979*a*). Cancer, ketosis and parenteral nutrition. *Med. J. Aust.*, **1**, 398.

CONYERS, R.A.J., NEED, A.G., ROFE, A.M., POTEZNY, N. & KIMBER, R.J. (1979*b*). Nutrition and Cancer. *Br. Med. J.* **i**, 1146.

CONYERS, R.A.J., ROFE, A.M., POTEZNY, N. & CARMAN, J.A. (1982). Ketotic metabolic states: studies with tumour-bearing mice ahd patients. *Int. Cong. Biochem.*, 123 (abstract).

GOODGAME, J.T., LOWRY, S.F., REILLY, J.J., JONES, D.C. & BRENNAN, M.F. (1979). Nutritional manipulations and tumour growth 1. The effects of starvation. *Am. J. Clin. Nutr.*, **32**, 2277.

KEPPLER, D. & DECKER, K. (1974). In *Methods in Enzymatic Analysis*, Bergmeyer, H.-U. (ed) **3**, 1127. Academic Press: New York and London.

KRALOVIC, R.C., ZEPP, E.A. & CENEDELLA, R.J. (1977). Studies on the depletion of carcass fat in experimental cancer. *Eur. J. Cancer*, **13**, 1071.

LUNDHOLM, K., EDSTROM, S., KARLBERG, I., EKMAN, L. & SCHERSTEN, T. (1980). Relationship of food intake, body composition, and growth to host metabolism in non-growing mice with sarcoma. *Cancer Res.*, **40**, 2516.

MAGEE, B.A., POTEZNY, N., ROFE, A.M. & CONYERS, R.A.J. (1979). The inhibition of malignant cell growth by ketone bodies. *Aust. J. Exp. Biol. Med. Sci.*, **57**, 529.

McGARRY, J.D., MEIER, J.M. & FOSTER, D.W. (1973). The effect of starvation and refeeding on carbohydrate and lipid metabolism *in vivo* and in the perfused rat liver. *J. Biol. Chem.*, **248**, 270.

MIDER, G.B. (1951). Some aspects of nitrogen and energy metabolism in cancerous subjects. *Cancer Res.*, **11**, 821.

ROBINSON, A.M. & WILLIAMSON, D.H. (1980). Physiological roles of ketone bodies as substrates and signals in mammalian tissues. *Physiol. Rev.*, **60**, 143.

ROFE, A.M. & WILLIAMSON, D.H. (1983). Metabolic effects of vasopressin infusion in the starved rat: Reversal of ketonaemia. *Biochem. J.*, **212**, 231.

SAUER, L.A. & DAUCHY, R.T. (1983). Ketone body, glucose, lactic acid and amino acid utilization by tumours *in vivo* from fasted rats. *Cancer Res.*, **43**, 3497.

SCHEIN, P.S., KISNER, D., HELLER, D., BLECHER, M. & HAMOSH, M. (1979). Cachexia of malignancy. Potential role of insulin in nutritional management. *Cancer*, **43**, 2070.

SHERWIN, R.S., HENDLER, R.G. & FELIG, P. (1975). Effect of ketone infusion on amino acid and nitrogen metabolism in man. *J. Clin. Invest.*, **55**, 1382.

STEIN, T.P. (1978). Cachexia, gluconeogenesis and progressive weight loss in cancer patients. *J. Theor. Biol.*, **73**, 51.

TISDALE, M.J. & BRENNAN, R.A. (1983). Loss of acetoacetate coenzyme A transferase activity in tumours of peripheral tissues. *Br. J. Cancer*, **47**, 293.